# Potential increase of the U.S. total fertility rate resulting from restorative treatment of unresolved subfertility: a simulation study

**DOI:** 10.3389/frph.2026.1856175

**Published:** 2026-06-16

**Authors:** J. B. Stanford, E. Harris, S. Najmabadi, K. R. Smith

**Affiliations:** 1 Office of Cooperative Reproductive Health, Division of Public Health, Department of Family Medicine and Public Health, Spencer Fox Eccles School of Medicine, University of Utah, Salt Lake City, UT, United States; 2 Kem C. Gardner Policy Institute, David Eccles School of Business, University of Utah, Salt Lake City, UT, United States; 3 Department of Family and Consumer Studies, University of Utah, Salt Lake City, UT, United States

**Keywords:** infertility, reproductive medicine, restorative reproductive medicine, subfertility, total fertility rate

## Abstract

**Background:**

The total fertility rate (TFR) in most developed countries has been declining for decades. In the United States (U.S.), the total fertility rate has remained below replacement level since 2007. Subfertility affects at least 15% of women or couples over their reproductive lifespan and contributes to reduced TFR. Restorative reproductive medicine (RRM) is a medically based approach to subfertility care that can be delivered in primary care settings to increase live birth rates.

**Objective:**

To estimate the theoretical impact of use of RRM among subfertile couples in the United States.

**Methods:**

We conducted a simulation study. Model inputs included the number of women of reproductive age in the United States by 5-year age groups; current age-specific and total fertility rates; the proportion of women in each age group with subfertility; estimated spontaneous live birth rates among women with subfertility; and age-specific crude live birth rates with RRM treatment. We evaluated fifteen scenarios including sensitivity analyses: two different varying assumptions for live births from spontaneous conception (25% vs. 50%), two levels of RRM utilization among subfertile women (20% vs. 50%), three different estimates of the number of subfertile women who would be potentially eligible for RRM treatment, and 4 different levels of effectiveness (live birth) from RRM treatment.

**Results:**

The baseline TFR in the United States was 1.77 during 2015-2019, and 13.5% of women ages 20-44 were estimated to have subfertility. In a conservative scenario (50% spontaneous births; 20% RRM utilization; married women trying to conceive for at least 12 months, 20.7% RRM live births), the TFR increased to 1.79, representing a 1.0% relative increase (absolute +0.02). In an optimistic scenario (25% spontaneous births; 50% RRM utilization; all subfertile women), the TFR increased to 2.02, a 14.5% relative increase (absolute +0.26), approaching replacement-level fertility.

**Conclusion:**

Simulation results suggest that expanding access to RRM within primary care settings could meaningfully increase the U.S. TFR, by reducing unresolved subfertility. Realizing this potential would require policy and health system changes to address workforce capacity, insurance coverage, and equitable access. These findings underscore the potential contribution of non-IVF fertility care pathways in addressing population-level fertility decline.

## Introduction

Birth rates have been declining rapidly in most countries worldwide, raising demographic and social concerns about shrinking population (1, 2). The total fertility rate (TFR) represents the sum of age-specific live birth rates among females in a given country or region at a specific point in time, providing an estimate of the average number of children per woman across the reproductive lifespan. A TFR of approximately 2.1 is required to maintain a stable population without migration (1). Globally, TFR continues to fall, leading to population decline in many countries (3). In the United States, TFR has generally been below replacement since 1971 and steadily below replacement since 2007; as of 2024, it stands at about 1.6 (1).

Many complex factors contribute to the decline in TFR, and it remains unclear which have the greatest influence (4). One important contributing factor is subfertility, when women/couples want a child but are unable to conceive or experience a prolonged delay (5, 6). Globally, the cumulative lifetime prevalence of subfertility among women/couples who have attempted to conceive is estimated at 8%-35%, with substantial regional variation (3). Subfertility may involve failure to conceive for a year or more of regular heterosexual intercourse without contraception (clinically termed “infertility”), and/or pregnancy loss prior to live birth, most commonly experienced as miscarriage in the first trimester. The U.S. National Survey of Family Growth (NSFG) uses a similar concept, termed impaired fecundity, defined as: (1) physical difficulty in becoming pregnant or carrying a pregnancy to term, (2) a period of 12 months or more of unsuccessful attempts to conceive, or (3) a physician-diagnosed condition that impairs the ability to conceive or carry a pregnancy to term. The NSFG assesses the proportion of women experiencing impaired fecundity by age groups; for example, the overall proportion of women 25-49 years old with impaired fecundity was 15.4% in 2015-2019 (6). In this paper, we use impaired fecundity from the NSFG as our primary measure of subfertility for a simulation study; in alternative scenarios, we also use impaired fecundity among married women, as well as the NSFG measure of “infertility,” which was married women who were trying to conceive for 12 months or more.

Subfertility may ultimately resolve with or without treatment; however, treatment increases the likelihood of resolution (7). Further, even if subfertility is eventually resolved through spontaneous conception, delays from lack of treatment may result in fewer children born than would have been desired by the woman or couple (8).

Options for treating subfertility include assisted reproductive technologies (ART), including hyperstimulation of the ovaries, oocyte retrieval, *in vitro* fertilization (IVF) with or without intracytoplasmic sperm injection (ICSI), and embryo transfer (9). While IVF and related procedures can help many couples conceive, they are costly and have barriers to access (10). For this reason, among others, non-IVF treatments are an important option for resolving subfertility (11, 12). Other treatments include intrauterine insemination (IUI), ovulation induction, and female and male reproductive surgeries. Currently in the United States, about 2% of births result from IVF, and an estimated 5% from non-IVF fertility treatment (13, 14).

An integrated non-IVF option for fertility treatment is restorative reproductive medicine (RRM), which includes identifying the fertile window of the menstrual cycle, addressing behavioral factors, and identifying and treating underlying diseases and conditions contributing to subfertility in both females and males (15–17). It also commonly includes ovulation induction and may include restorative reproductive surgery (18). Primary care clinicians can be trained to provide RRM (excluding surgery), which has strong potential for expanding access to fertility care (19).

We conducted a simulation study, using data from the United States (7 centers) to estimate the potential impact on TFR if women/couples with subfertility had ready access to RRM. We employed a range of scenarios in reference to different hypothetical levels of utilization and for expected spontaneous birth rates, and birth rates with RRM treatment, based on available data.

## Materials and methods

### Study population

For this simulation study, the study population was women ages 20-44 in the United States. To estimate the potential impact of RRM treatment, we used data from an international multicenter study of RRM treatment, the international Natural Procreative Technology Evaluation and Surveillance of Treatment (iNEST) study (20). Couples in this study sought treatment to have a live birth, and received RRM evaluation and medical treatments. About half of iNEST participants were from the U.S., the remainder were from Canada, the U.K., and Poland.

### Statistical analyses

#### Age specific fertility rates, and numbers of women with subfertility

We conducted a simulation study across fifteen different specific scenarios. From the Census Bureau's American Community Survey, we obtained the number of women of reproductive age in the United States in 2015-2019, divided into 5-year age bands: 20-24, 25-29, 30-34, 35-39, and 40-44. For the same age bands and years, we also calculated the average age-specific birth rates, which sum to the TFR (21). Next, from the NSFG, we obtained estimates in 2015-2019 of impaired fecundity for the age bands 15-24, 25-29, 30-34, 35-39, and 40-44 years (6). We did not include ages 15-19, or ages 45-49 in our model, because fertility in these age groups contributes minimally to the TFR, and also because RRM is not a viable option for females of age 45 or more. We multiplied the age-specific rates of impaired fecundity by the number of women in each age stratum, to obtain the number of women with impaired fecundity (“subfertility”) in each age stratum. For additional scenarios, we similarly calculated the number of women impaired fecundity who were married, in each age stratum (“married subfertility”). Based on another measure within the NSFG, we also calculated the number of women who were married and actively trying to conceive for at least a year (“married infertility”). Finally, we created scenarios where we varied the live birth rate (success rate) for RRM.

#### Spontaneous resolution of subfertility

In subfertile women or couples, reported spontaneous pregnancy and live birth rates vary considerably (20%–60%), influenced by follow-up period and factors such as female age and duration of subfertility (22–26). For example, in a regional study in Scotland of 1,316 couples ages 18–50 with unexplained subfertility, the natural conception rate within one year leading to subsequent live birth was 24% (25). In another regional study in New Zealand, 1,386 infertile couples with no restriction on female age were followed for a median of 13 years, with a cumulative live birth rate of 62% (24). For our models, we chose parameters of spontaneous live births occurring in either 25% or 50% of those who would have been potentially eligible for RRM treatment. In the model, these spontaneous live births are removed from eligibility for RRM treatment, and thus cannot contribute to a projected increase in TFR.

#### RRM treatment resolution of subfertility (live births with RRM treatment)

We used birth within 18 months of entry to the study, stratified by woman's age at the start of the study, using the same age strata as noted above. The denominator included all women who entered the study within each age stratum, and the numerator all live births that occurred through 18 months of follow-up. Births following reproductive surgeries (e.g., female laparoscopy) were excluded from this simulation, to focus on medical interventions that can be implemented by primary care clinicians. The overall live birth rate from RRM in the iNEST study, excluding surgeries was 20.7% (see Table 1).

**Table 1:** Age-specific crude live birth rates from the international natural procreative technology surveillance of treatment (iNEST) study (medical treatment only).

Woman's age in years at start of treatment	Crude proportion live births within 1.5 Years
20–24	0.235
25–29	0.237
30–34	0.214
35–39	0.217
40–44	0.135
All ages	0.207

While no other RRM studies have published age-stratified crude birth rates, we can compare the iNEST overall crude live birth rate with other single-center studies of RRM. Studies of RRM for subfertile couples with follow-up to 24 months have reported crude live birth rates as follows: Ireland (2008) 26% (27), Canada, 38% (28), USA, 18% (19), Spain 35% (29), Ireland (2019) 41% (30). We therefore conducted additional sensitivity analyses using overall RRM crude birth rates of 18%, 35%, and 41%.

#### Ethical approval

This study is a simulation study that used publicly accessible data. We also used de-identified data from the iNEST cohort study, which was approved by the University of Utah Institutional Review Board, IRB #00014070.

## Results

The RRM age-specific crude birth rates from the iNEST study, excluding surgical treatments, are reported in Table 1. We report the cumulative birth rates through 18 months of follow-up.

To assess the potential impact of the use of RRM by subfertile couples, we modeled fifteen simulated scenarios based on a range of parameters, summarized in Tables 2–5. In the baseline scenario, which reflects U.S. data from 2015 to 2019, the TFR was 1.77 prior to any simulated interventions. In our initial simulated scenario for the impact of broad access to RRM treatment (Table 2, Scenario 1), we assumed that 50% of subfertile couples would achieve a live birth without any treatment, which would ultimately contained within the TFR. Additionally, we assumed that 20% of the remaining subfertile population utilize RRM treatment. Assuming that the women potentially eligible for RRM treatment were all of the subfertile women in the USA yielded a 3.9% relative increase in the TFR, corresponding to an absolute increase of 0.07 and a projected TFR of 1.84 (Table 2, scenario 1). Using the same assumptions, but restricting eligibility for RRM treatment to women who were married yielded a 1.8% relative increase in the TFR, corresponding to an absolute increase of 0.03 and a projected TFR of 1.80 (Table 3, scenario 5). Further restricting eligibility for RRM treatment to married women who have been trying to conceive for at least 12 months yielded a 1.0% relative increase in the TFR, corresponding to an absolute increase of 0.02 and a projected TFR of 1.79 (Table 4, scenario 9).

**Table 2:** Model input assumptions and projected increases in total fertility rate (TFR) from baseline, using U.S. data, based on total women with impaired fecundity receiving RRM, 2015–2019. Models vary the proportion with spontaneous live birth and the proportion using RRM treatment, while keeping constant the RRM live birth rate.

Scenario	Proportion spontaneous live birth	Proportion using RRM treatment	Overall RRM live birth rate	TFR	Absolute increase from baseline	Relative increase from baseline
baseline	na	0%	NA	1.77	0.00	0%
1	50%	20%	20.7%	1.84	0.07	3.9%
2	25%	20%	20.7%	1.87	0.10	5.8%
3	50%	50%	20.7%	1.94	0.17	9.7%
4	25%	50%	20.7%	2.02	0.26	14.5%

**Table 3:** Model input assumptions and projected increases in total fertility rate (TFR) from baseline, using U.S. data, based on married women with impaired fecundity receiving RRM, 2015–2019. Models vary the proportion with spontaneous live birth and the proportion using RRM treatment, while keeping constant the RRM live birth rate.

Scenario	Proportion spontaneous live birth	Proportion using RRM treatment	Overall RRM live birth rate	TFR	Absolute increase from baseline	Relative increase from baseline
baseline	na	0%	NA	1.77	0.00	0%
5	50%	20%	20.7%	1.80	0.03	1.8%
6	25%	20%	20.7%	1.82	0.05	2.8%
7	50%	50%	20.7%	1.85	0.08	4.6%
8	25%	50%	20.7%	1.89	0.12	6.9%

**Table 4:** Model input assumptions and projected increases in total fertility rate (TFR) from baseline, using U.S. data, based on married women with infertility and receiving RRM, 2015–2019. Models vary the proportion with spontaneous live birth and the proportion using RRM treatment, while keeping constant the RRM live birth rate.

Scenario	Proportion spontaneous live birth	Proportion using RRM treatment	Overall RRM live birth rate	TFR	Absolute increase from baseline	Relative increase from baseline
baseline	na	0%	NA	1.77	0.00	0%
9	50%	20%	20.7%	1.79	0.02	1.0%
10	25%	20%	20.7%	1.79	0.03	1.5%
11	50%	50%	20.7%	1.81	0.04	2.5%
12	25%	50%	20.7%	1.83	0.07	3.7%

**Table 5:** Model input assumptions and projected increases in total fertility rate (TFR) from baseline, using U.S. data, based on married women with impaired fecundity receiving RRM, 2015–2019. Models assume proportion with a spontaneous live birth 50% and proportion using RRM treatment 50%, with a variable live birth rate for RRM treatment. (Scenario 7 in this table is the same as scenario 7 in Table 3.).

Scenario	Proportion spontaneous live birth	Proportion using RRM treatment	Overall RRM live birth rate	TFR	Absolute increase from baseline	Relative increase from baseline
baseline	na	0%	NA	1.77	0.00	0%
13	50%	50%	18.0%	1.84	0.07	4.0%
7	50%	50%	20.7%	1.85	0.08	4.6%
14	50%	50%	35.0%	1.90	0.14	7.8%
15	50%	50%	41.0%	1.93	0.16	9.1%

In a more optimistic simulated scenario (Table 2, Scenario 4), we assumed that the proportion of subfertile couples experiencing a live birth without treatment was 25%, and that 50% of the remaining subfertile population utilize RRM treatment. In this scenario, the TFR had a 14.5% relative rise and a 0.26 absolute rise, yielding a projected TFR of 2.02. Using the same assumptions, but restricting potential eligibility for RRM treatment to women who were married yielded a 6.9% relative increase in the TFR, corresponding to an absolute increase of 0.12 and a projected TFR of 1.89 (Table 3, scenario 8). Further restricting eligibility for RRM treatment to married women who have been trying to conceive for at least 12 months yielded a 3.7% relative increase in the TFR, corresponding to an absolute increase of 0.07 and a projected TFR of 1.83 (Table 4, scenario 12).

**Figure 1:**
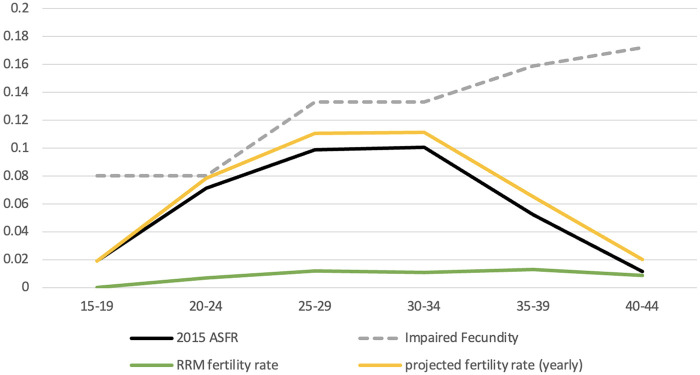
Age-specific fertility rates (ASFR), proportions with impaired fecundity, restorative reproductive medicine (RRM) age-specific fertility rates, and projected (modeled) total age- specific fertility rates, based on Table 2, Scenario 4.

**Figure 2:**
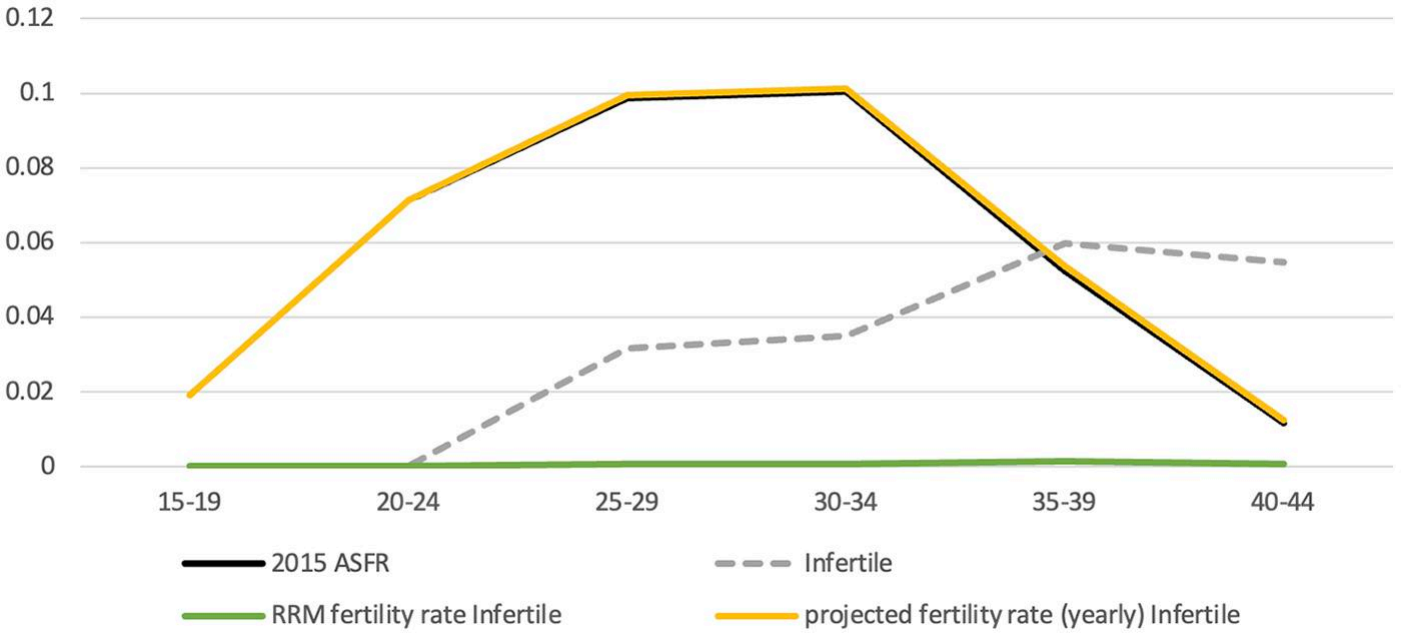
Age-specific fertility rates (ASFR), proportions with married infertility, restorative reproductive medicine (RRM) age-specific fertility rates, and projected (modeled) total age- specific fertility rates, based on Table 4, Scenario 9.

Figures 1, 2 show the contributions of each age-specific model input to the age-specific fertility rates (components of the TFR) for the most optimistic scenario (Table 2, Scenario 4) and the most conservative scenario (Table 4, Scenario 9), respectively. Intermediate scenarios are also summarized in Tables 2–4.

Finally, in additional sensitivity analyses, we used an intermediate scenario of 50% spontaneous live birth followed by 50% RRM utilization, and varied the assumption of RRM effectiveness from 18% to 41% (based on available studies of RRM). This yielded absolute increases in the TFR ranging from 0.02 to 0.07 (projected TFR of 1.84–1.93), and a relative increase in TFR from 4.0% to 9.1%.

## Discussion

Our simulation study indicates that expanding access to RRM-based fertility treatment could meaningfully reduce unresolved subfertility and potentially shift the TFR toward the replacement level. Since this simulation represents a hypothetical nationwide shift in medical practice, it is important to consider the practical and logistical challenges that would be required to make RRM accessible and available. In addition, factors that could influence actual utilization and potential alternative interventions should also be addressed.

One major consideration for ensuring the availability of RRM is determining who will provide these services and what level of training they will require. Primary care clinicians could play a pivotal role in delivering RRM, given their accessibility and ongoing patient relationships. However, this potential is constrained by a general shortage of primary care personnel in the United States, across all types of primary care clinicians (31–33). The time and resources required for training can also be a limiting factor, and certification pathways are currently not universally standardized. However, there are several sources of postgraduate training in RRM currently available in the U.S., which could be expanded (34). In addition, achieving full RRM capacity and potential likely requires the training and involvement of fertility care educators and possibly other allied health professionals, such as health coaches or fertility educators (35).

Besides the availability of clinicians and health personnel, coverage must also be accessible and affordable. RRM care overlaps with care for general health conditions, including hormonal or metabolic health, such as polyendocrine metabolic ovarian syndrome or hypothyroidism (36, 37). However, some fertility specific treatments are also involved, such as ovulation induction, necessitating insurance coverage specific for fertility treatment. Currently, insurance coverage for fertility treatment varies widely across the United States, with 20 states mandating insurance coverage as of 2022, under which the full spectrum of RRM service should be covered (38–40).

In addition to access to care, both logistically and financially, other factors and barriers influence utilization of fertility care (8). A key factor is providing culturally sensitive care (41–43). RRM can expand access to care to populations for whom IVF and related procedures are not acceptable (44). There is inherent complexity of treatment decisions that involves financial, social, cultural, and ethical factors, and their impacts on the persons involved (45). It is also important to keep in mind that concerns for population health need to encompass a broader perspective of quality of life and human flourishing, rather than focusing solely on birth rates (46).

As an alternative option to RRM, it would also be possible to raise the TFR by increasing utilization of more procedural forms of fertility care, such as IVF; however, the supply of physicians providing this type of care is limited (47), which is one reasons that the majority of births related to medical fertility treatment in the United States are from non-IVF approaches (14, 38, 48). From a social policy perspective, we believe that prioritizing initial access to non-IVF fertility care, including systemic approaches like RRM, appears reasonable, doing it in a way that expands access, and does not limit options including accessibility to IVF (49). Simulation studies and population-based data suggest that initiating fertility treatment within a primary care setting before progressing to IVF is beneficial and can reduce utilization of IVF without lowering ultimate birth rates (50, 51). Faciliting additional treatment of subfertility in primary care settings could also make IVF more accessible to patients who need it in order to achieve a birth. Under the principal of patient autonomy, patients who would be able to conceive with RRM may still choose to pursue IVF treatment directly. Also essential are systematic efforts to track and improve all types of fertility care (11).

Limitations of our study include the exclusion of surgery, an essential restorative intervention for some couples using RRM (18). Our results, therefore, are based on pregnancy rates without surgical treatment, yet they still suggest that medically-based RRM can make a substantial contribution to fertility care.

Another limitation is that participants in the iNEST study, from which birth rates for RRM were drawn, differ from the population of the United States in ways that can impact utilization and outcomes of fertility treatment. Virtually all iNEST patients were married, and they had a high level of education. Specifically, among women participating in iNEST with educational data, 48.3% had 16-18 years of education and 31.6% had over 18 years of education (20). Among married women with impaired fecundity in the NSFG, 26.4% had a bachelor's degree, and 16.4% had a master's degree or higher [calculated from Table 3 in (6)]. Parity was more comparable: 37.8% of iNEST women had no prior births, while 29.8% had a prior birth among married women with impaired fecundity in the NSFG.

In this regard, it is relevant to note that while utilization of all fertility treatment, including IVF, is substantially higher among persons with higher income and higher education, multiple social and cultural factors influence whether fertility treatment of any kind is actually used (52–54). Fertility policy in the United States varies at the state and federal levels and influences coverage, accessibility, and affordability (40). RRM has a potential to help expand access for affordability and for cultural acceptability (55, 56).

An additional caution in considering our model is that fertility rates are changing rapidly, and the current prevalence of subfertility may differ substantially from estimates of impaired fecundity in the NSFG data (2015-2019). The actual desire for fertility treatment was also not a parameter available for our model. Even when desire is measured, it does not necessarily translate to intention and behavior to seek to conceive, or to seek fertility treatment (45, 57). Finally, our model simulates potential changes in TFR at a single point in time, and it remains unclear whether such changes could be sustained over the long term.

Strengths of our simulation study include the use of population-based data to estimate the number of women with subfertility, as well as age-stratified data from a multi-site study on RRM fertility outcomes with primary care clinicians, excluding surgical treatments (20).

Unresolved subfertility not only burdens couples but also contributes to societal challenges associated with a TFR below replacement, including significant social and economic implications. RRM offers a potential cost-effective strategy to help alleviate these burdens. With appropriate caveats, our simulation study suggests that expanding the availability and utilization of RRM could have a measurable impact on increasing the TFR in the United States. We emphasize that these results are theoretical, are based on several modeling assumptions, and require additional empirical studies to validate the predictions. Whether RRM would be more widely utilized if made broadly accessible remains an open question. Future research, including longitudinal studies, should further explore patients’ actual treatment choices in settings where a spectrum of options is available and use real-world data to formally evaluate the effectiveness, cost and cost-effectiveness of RRM at a population level. Pilot implementation studies and comparative effectiveness studies would be valuable next steps to collect empirical data to evaluate the potential clinical and population benefits of increasing access to and utilization of RRM care.

## Data Availability

The raw data supporting the conclusions of this article will be made available by the authors, without undue reservation.
